# Advances in diagnosis and treatment of lung cancer-associated obstructive pneumonia and lung abscess: synergistic strategies for infection control and antitumor therapy

**DOI:** 10.3389/fcimb.2025.1638997

**Published:** 2025-10-14

**Authors:** Peijun Cao, Li Chen, Shaohua Xie, Bin Hu

**Affiliations:** Department of Thoracic Surgery, Sichuan Clinical Research Center for Cancer, Sichuan Cancer Hospital & Institute, Sichuan Cancer Center, University of Electronic Science and Technology of China, Chengdu, China

**Keywords:** advanced lung cancer, obstructive pneumonia, pulmonary abscess, nanomedicine, antimicrobial therapy

## Abstract

Lung cancer-associated obstructive pneumonia and pulmonary abscess represent critical oncologic complications. Effective management hinges on dynamically balancing primary tumor control with secondary infection management. Recent major advances in targeted therapy, immunotherapy, and novel anti-infective technologies have driven a shift towards integrated, multidimensional clinical strategies.This review details diagnostic and therapeutic progress for these conditions, emphasizing the critical balance between controlling severe infections and managing antitumor therapy toxicities, alongside recent advances in nanomedicine for pulmonary infections.

## Introduction

Lung cancer remains the leading cause of cancer-related mortality worldwide. According to the latest statistics from the China National Cancer Center (2022), approximately 1,060,600 new lung cancer cases were diagnosed, accounting for 22.0% of all malignant tumors, with roughly 733,300 deaths (Zheng et al., 2024). Notably, advanced lung cancer often causes bronchial obstruction due to tumor progression, leading to distal lung parenchymal infection, termed post-obstructive pneumonia (POP). POP incidence reaches 45-55%, with 10-15% of cases progressing to pulmonary abscess (Valvani et al., 2019; Moretti et al., 2023). Central lung cancer, particularly squamous cell carcinoma, exhibits a significantly higher incidence of obstructive pneumonia than peripheral types. This results from bronchial obstruction and impaired mucociliary clearance, leading to secretions retention and bacterial colonization (Wei et al., 2024). Furthermore, the immunosuppressive state in advanced lung cancer patients may substantially elevate the risk of pulmonary abscess formation.

Postobstructive pneumonia exerts a significant adverse impact on the quality of life and prognosis of lung cancer patients. Studies indicate that patients with concurrent postobstructive pneumonia often experience severe limitations in daily activities due to persistent dyspnea, recurrent fever, and purulent sputum production. Frequent hospitalizations and prolonged use of broad-spectrum antibiotics further contribute to treatment-related complications (e.g., nephrotoxicity, antibiotic-associated diarrhea) while concurrently impeding the delivery of antitumor therapies (Jo, 2022). A study revealed that patients with stage I/II lung cancer comorbid with chronic obstructive pulmonary disease (COPD) were less likely to undergo surgery (56.8% vs. 65.9%) or surgery with adjuvant chemotherapy (15.4% vs. 17.1%), but more likely to receive radiotherapy (26.0% vs. 21.8%) (all p<0.001). Among stage III/IV patients, those with COPD showed lower rates of chemotherapy (55.9% vs. 64.4%) or radiotherapy (42.5% vs. 47.5%) (all p<0.001) (Goffin et al., 2021). Current evidence primarily consists of sporadic retrospective studies with limited sample sizes. A retrospective analysis of 408 stage III/IV lung cancer patients revealed a 30-day mortality rate of 30% in those with obstructive pneumonia. Multivariate logistic regression identified CURB-65 score (OR: 73.20, p = 0.001) and smoking status (OR: 0.009, p = 0.015) as significant predictors of 30-day mortality (Moretti et al., 2023). Conversely, a separate study on surgico-pathological features of superficial endobronchial lung cancer (SELC) reported no significant prognostic impact (P = 0.96) from tumor-associated obstructive atelectasis or pneumonia (Chen et al., 2010). Therefore, it remains challenging to draw definitive conclusions regarding whether obstructive pneumonia or lung abscess impacts the prognosis of lung cancer. Prospective studies are warranted to address this clinical question. Furthermore, existing research has demonstrated the specific pro-tumorigenic role of inflammation in lung cancer progression. Notably, IL-6 has been identified as a key mediator that facilitates both tumor initiation and advancement, thereby establishing a vicious cycle (Ochoa et al., 2011; Chen et al., 2020). Based on the evidences, infectious complications should be considered as an independent prognostic factor in lung cancer management strategies, with early intervention demonstrating potential to enhance quality of life and prolong survival.

## Pathophysiological mechanisms

**Figure 1:**
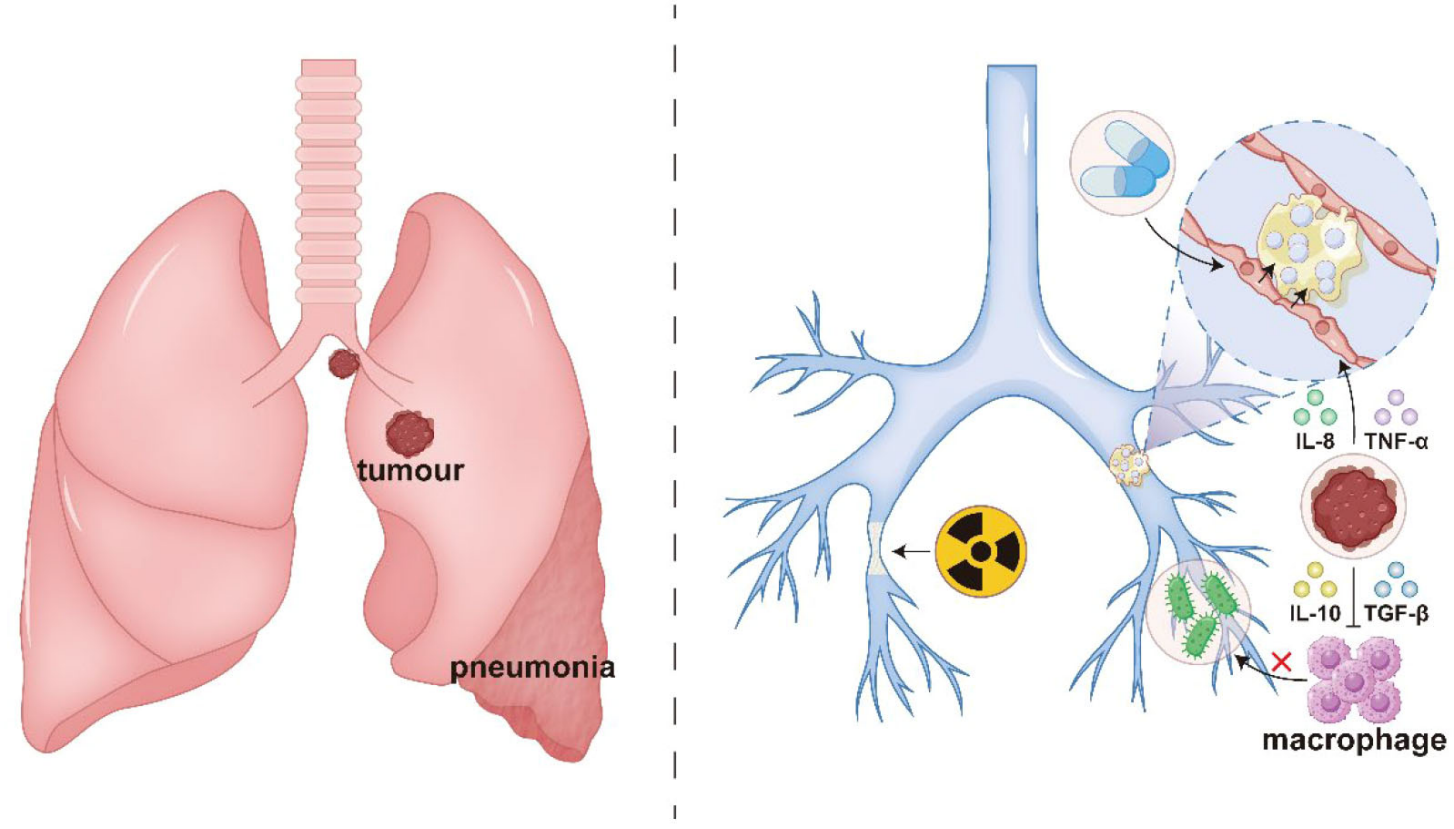
Direct and indirect mechanisms in the pathogenesis of obstructive pneumonia: tumor-induced bronchial stenosis and obliteration (direct); and tumor-associated inflammation and immune dysregulation (indirect).

The direct mechanisms of bronchial obstruction caused by lung cancer primarily involve tumor-related mechanical compression or infiltration: centrally located tumors (e.g., squamous cell carcinoma or small cell carcinoma) may grow intraluminally, resulting in bronchial stenosis or complete occlusion, while extrinsic compression from mediastinal lymph node metastases can similarly obstruct airways, ultimately leading to distal atelectasis (Moretti et al., 2023). Indirect mechanisms involve tumor-associated inflammation and immune dysregulation: The tumor microenvironment secretes pro-inflammatory factors (e.g., IL-8, TNF-α), inducing bronchial mucosal edema and mucus hypersecretion, which leads to mucus plug formation and exacerbates airway obstruction (Oliviero et al., 2016; Cazzola et al., 2017; Ragnoli et al., 2024). Concurrently, immunosuppressive factors (e.g., IL-10, TGF-β) impair local macrophage function, increasing susceptibility to opportunistic infections (e.g., Pseudomonas aeruginosa or Aspergillus) (Meziani et al., 2021; Wei et al., 2022; Cui et al., 2023). Subsequent infectious inflammation further disrupts airway architecture. Treatment-related injuries also contribute to indirect obstructive mechanisms, such as post-radiotherapy fibrosis causing loss of bronchial elasticity (Ozkok et al., 2008),while chemotherapeutic agents (e.g., gemcitabine) may induce mucositis, exacerbating cicatricial stenosis (Lee et al., 2008), as illustrated in **Figure 1**.

## Clinical manifestations and differential diagnosis

Clinical presentation and differential diagnosis of lung cancer-associated obstructive pneumonia require integrated analysis of symptoms, imaging, and microbiological features.Obstructive Pneumonia: Characterized by persistent cough, purulent sputum, fever (>38.5°C), and dyspnea. High-resolution CT typically shows localized consolidation with proximal bronchial obstruction or mucus plugging.Pulmonary Abscess: Presents with high fever (>39°C) and copious foul-smelling purulent sputum (producing 300–500 mL/day). CT reveals thick-walled cavities (>2 cm diameter) with air-fluid levels and surrounding inflammatory infiltration.Elevated infectious biomarkers (e.g., Procalcitonin/PCT, C-reactive Protein/CRP) and positive microbiological identification of pathogens (e.g., Klebsiella pneumoniae, Pseudomonas aeruginosa) in respiratory samples (sputum/bronchoalveolar lavage fluid) support the diagnosis of bacterial pneumonia while ruling out alternative causes. The differential diagnosis should encompass the following conditions:Infectious pneumonia: Non-obstructive pneumonia typically presents with multifocal distribution, a broader spectrum of pathogens (e.g., viruses, Mycoplasma), and no direct evidence of tumor compression.Radiation pneumonitis: Confined to the radiation field, early-stage manifestations include ground-glass opacities with traction bronchiectasis, progressing to fibrosis in later stages, and absence of purulent sputum.Immune checkpoint inhibitor (ICI)-associated pneumonitis: Predominantly manifests as interstitial or organizing pneumonia, with histopathological findings of CD8+ T-cell infiltration and temporal association with ICI administration (Zhang et al., 2021), (**Table 1**). Pulmonary tumor metastasis: Characterized by multiple nodules or diffuse miliary lesions without elevated infectious inflammatory markers (Oliaro et al., 2008). Additionally, metagenomic next-generation sequencing (mNGS) of bronchoalveolar lavage fluid (BALF) enhances detection rates for mixed infections (e.g., bacterial-fungal coinfection) (Jiang et al., 2024), while bronchoscopic biopsy can exclude tumor progression or foreign body obstruction.

**Table 1:** Differentiation and management of lung cancer-associated obstructive pneumonia/abscess versus immune checkpoint inhibitor pneumonia (CIP).

	Obstructive pneumonia/abscess	Immune checkpoint inhibitor pneumonia (CIP)
Etiology and Pathogenesis	Neoplastic airway obstruction complicated by bacterial/fungal superinfection	ICI-triggered T-cell-mediated pulmonary tissue damage
Imaging differentials	- Focal pulmonary consolidation with proximal bronchial cutoff and mucoid impaction - May develop cavitary changes	- Bilateral diffuse ground-glass opacities (GGO) or organizing pneumonia (OP)-pattern changes - Occasional interlobular septal thickening - Absence of demonstrable obstructive pathology
Laboratory and Etiologic Testing	- Elevated CRP/PCT levels -Pathogen detection (e.g., Klebsiella pneumoniae)	- No evidence of infection - Peripheral blood IL-6 or KL-6 levels may be elevated
Therapeutic Approaches	- Discontinuation of antineoplastic agents - Priority management of airway obstruction (surgical intervention/stent placement) - Antimicrobial Therapy: β-lactamase inhibitor combinations/carbapenems - Interventional drainage (bronchoscopic or percutaneous)	- ICI withdrawal - Mild cases (Grade 1): Prednisone 0.5–1 mg/kg/day orally - Severe cases (Grade 2-4): Intravenous methylprednisolone 1–2 mg/kg/day - Refractory cases may require adjunctive infliximab or mycophenolate mofetil.
Dynamic Monitoring	- Focus on etiological eradication (repeat cultures/PCT monitoring) - Assessment of tumor control (radiologic follow-up)	- Follow-up high-resolution CT and pulmonary function testing - Assessment of corticosteroid therapy response

## Current therapeutic strategies

At present, the treatment of lung cancer-related obstructive pneumonia and lung abscess mainly combines anti-infection treatment with non-anti-infection treatment. Non-anti-infection treatment strategies include relieving airway obstruction, surgical resection and local thoracic drainage, enhancing airway clearance, oral hygiene care, preventing reflux and aspiration, symptomatic supportive treatment, oxygen therapy and respiratory support, etc. It should be noted that if pneumonia occurs during tumor treatment, it is recommended to suspend anti-tumor treatment. Anti-tumor treatment can be considered to be restarted after the patient’s acute infection symptoms have completely resolved.

Research on the microbiological characteristics of patients with lung cancer complicated by obstructive pneumonia is currently limited. However, existing evidence indicates that the infectious microorganisms in these patients show significant diversity, mainly influenced by tumor-related immune deficiencies and treatment intervention factors (Zhao et al., 2023). The pathogen spectrum shows the coexistence of multiple pathogens, including bacteria, viruses and fungi (Meng et al., 2023; Shin et al., 2023). In patients who have not received systemic treatment and whose immune function is relatively intact, the main pathogenic bacteria are similar to those found in community-acquired pneumonia, mainly including Streptococcus pneumoniae, Haemophilus influenzae, Moraxella catarrhalis and respiratory viruses (Yamada et al., 2010). However, for patients receiving multi-line treatment (especially those in the advanced stage with induced multiple immunosuppression), there is a phenomenon of opportunistic pathogen colonization. The detection rates of Pseudomonas aeruginosa, Staphylococcus aureus, Enterobacter cloacae, and Acinetobacter species have significantly increased. It is worth noting that such patients often have concurrent oral anaerobic bacterial infections (such as Bacteroides species, Peptostreptococcus species, Fusobacterium species, and Actinomyces species) (Dong et al., 2021). Furthermore, the patient’s immune status plays a decisive role in the composition of pathogens: patients with a compromised cellular immune system are prone to concurrent infections by cytomegalovirus/herpesvirus and bacteria (Suri et al., 2024); Humoral immunosuppression significantly increases the risk of invasive fungal infections (including Candida, Aspergillus, Histoplasma, and Coccidioides species) and Pneumocystis jirovecii pneumonia. Consequently, initial antibiotic selection should typically cover common Gram-negative bacteria and include anaerobic activity. Suitable options include β-lactam/β-lactamase inhibitor combinations (e.g., amoxicillin/clavulanate 1.2 g every 8 hours, piperacillin/tazobactam 4.5 g every 8 hours, or cefoperazone/sulbactam 3.0 g every 8 hours). Subsequently, therapy should be refined based on microbiological identification and antimicrobial susceptibility testing results. We summarize recommended antimicrobial regimens for specific types of infectious pneumonia requiring particular attention in **Table 2**.

**Table 2:** Treatment recommendations for specific types of infectious pneumonia.

Classification	Name	Therapeutic approaches
Bacteria	MRSA	Vancomycin or linezolid; alternative regimens include daptomycin or telavancin (Liu et al., 2013)
	ESBL-producing Enterobacteriaceae	Ceftazidime/avibactam or meropenem plus amikacin (Wang et al., 2023)
	Carbapenem-resistant Pseudomonas aeruginosa	Ceftazidime/avibactam (if susceptible to the pathogen) or polymyxin B plus fosfomycin or high-dose doripenem (Doi, 2019)
	Carbapenem-resistant Acinetobacter baumannii	Polymyxin B plus high-dose sulbactam or tigecycline (Bartal et al., 2022)
Fungus	Aspergillus	Voriconazole; for resistance or intolerance, use isavuconazole or liposomal amphotericin B (Maertens et al., 2016).
	Mucor	Combined surgical debridement plus liposomal amphotericin B; followed by maintenance with isavuconazole or posaconazole (Bassetti and Bouza, 2017).
	Pneumocystis	Co-trimoxazole or caspofungin (Cushion and Walzer, 2009).
Virus	Influenza virus	Oseltamivir or baloxavir (Kumar et al., 2020)
	SARS-CoV-2	Nirmatrelvir/ritonavir or remdesivir (Li et al., 2023).
	CMV	Intravenous ganciclovir or valganciclovir (Kotton, 2013).

Non-anti-infective therapy centers on: 1. relieving airway obstruction via bronchoscopic interventions—including electrocautery, argon plasma coagulation, laser therapy, radiotherapy, and cryotherapy—as well as stent placement or mechanical debridement to restore ventilation, supplemented with aerosolization, postural drainage, and chest physiotherapy to promote airway clearance (Steinfort et al., 2021; Yang et al., 2024). 2.Surgical and local interventions: For refractory lung abscesses (failed medical therapy >6 weeks, cavity >6 cm, or suspected malignancy) or acute complications (massive hemoptysis, sepsis), perform lobar/wedge resection. Combine with imaging-guided percutaneous drainage(for peripheral abscesses) (Lee et al., 2022) or bronchoscopic drainage(for central abscesses) (Herth et al., 2005). 3.Stage-directed management of empyema: Conservative therapy for acute exudative phase; video-assisted thoracoscopic debridement for fibrinopurulent phase; decortication required in chronic phase. 4.Supportive care: Elevation of the head ≥30° to prevent aspiration, maintenance of fluid/electrolyte balance and nutritional support, with early fluid resuscitation for hypotension.5.Respiratory support: Select nasal cannula/facemask oxygen (for SpO_2_ ≥90% or 88%-92% with hypercapnia risk), prioritizing high-flow humidified oxygen therapy or non-invasive ventilation to reduce intubation rates.Therapy must balance antitumor and anti-inflammatory requirements, optimizing individualized regimens through minimally invasive techniques (navigational bronchoscopy, targeted ablation) and ongoing reassessment of infection-tumor interactions.

**Figure 2:**
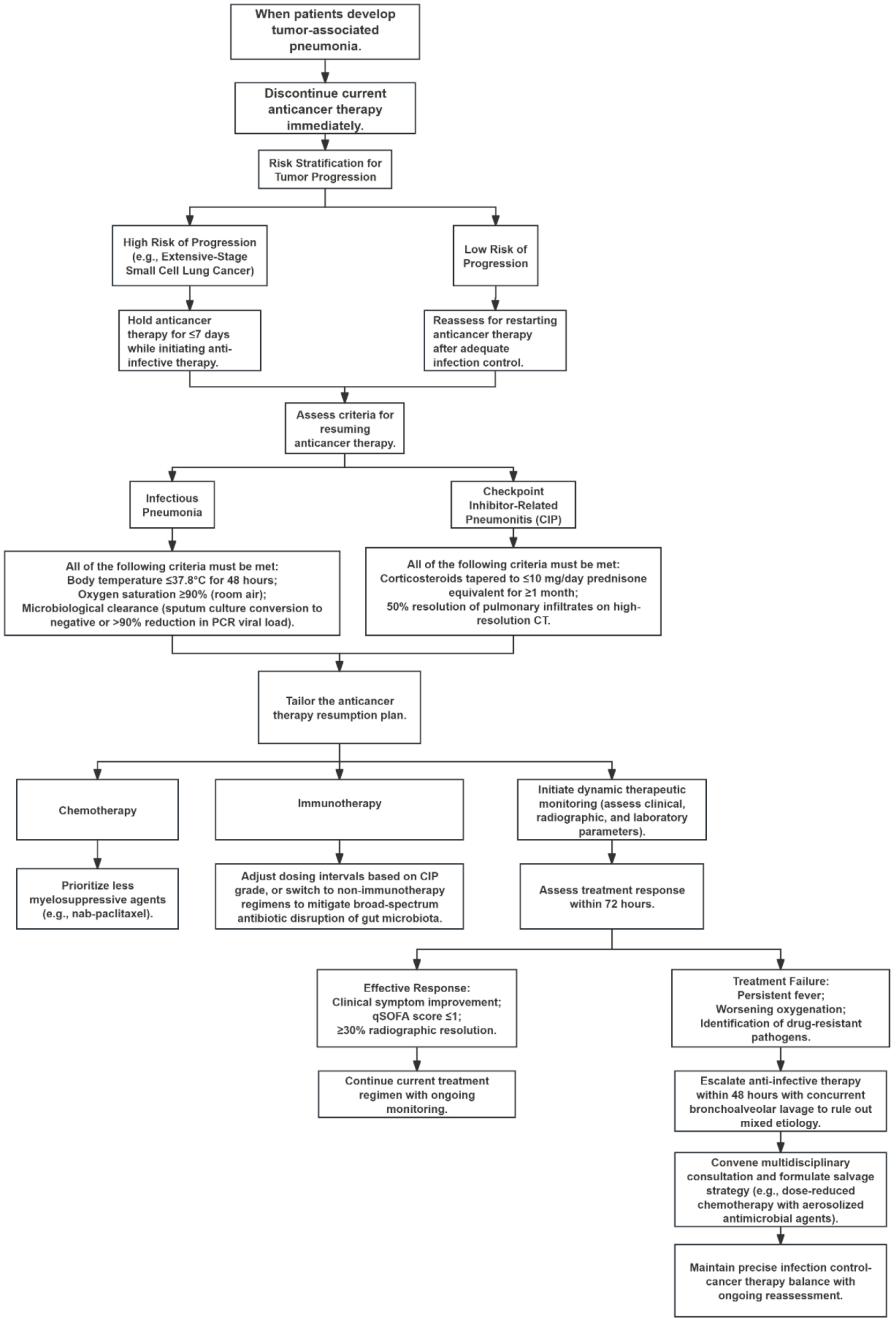
Treatment algorithm for tumor-associated pneumonia.

In managing tumor-associated pneumonia, the primary principle is stratified decision-making regarding antineoplastic therapy interruption and resumption, based on pneumonia type and cancer progression risk. Upon pneumonia onset, immediately suspend current antineoplastic regimens: For high-progression-risk malignancies (e.g., extensive-stage SCLC), limit interruption to ≤7 days with concurrent antimicrobial initiation; for low-risk tumors, defer resumption until infection control is achieved. Antineoplastic therapy restart requires strict clinical and laboratory criteria: For infectious pneumonia, patients must maintain temperature ≤37.8°C for 48 hours, SpO_2_; ≥90% on room air, and microbiological clearance (negative sputum culture or >90% reduction in PCR load) (Baden et al., 2012). For checkpoint inhibitor pneumonia (CIP), glucocorticoid tapering to prednisone <10 mg/day must be maintained for ≥1 month, with concurrent >50% infiltrate resolution on high-resolution CT. Regimen adjustments should prioritize: chemotherapy agents with minimal myelosuppression (e.g., nab-paclitaxel) (Chen Y. et al., 2022), immunotherapy modification via extended dosing intervals or transition to non-immunotherapeutic agents based on CIP grade; and avoidance of broad-spectrum antibiotics to prevent gut microbiota disruption. Therapeutic efficacy requires dynamic monitoring of clinical, radiological, and laboratory parameters: Improvement within 72 hours (clinical symptom resolution, qSOFA score ≤1, and ≥30% radiographic absorption) indicates treatment response. For persistent fever, worsening oxygenation, or detected drug-resistant pathogens, escalate antimicrobial therapy within 48 hours. Perform bronchoscopic alveolar lavage to exclude mixed etiologies and convene multidisciplinary consultation to establish salvage strategies (e.g., dose-reduced chemotherapy with aerosolized antimicrobials), achieving precision balance between infection control and oncologic management, as illustrated in **Figure 2**.

## Case presentation

**Figure 3:**
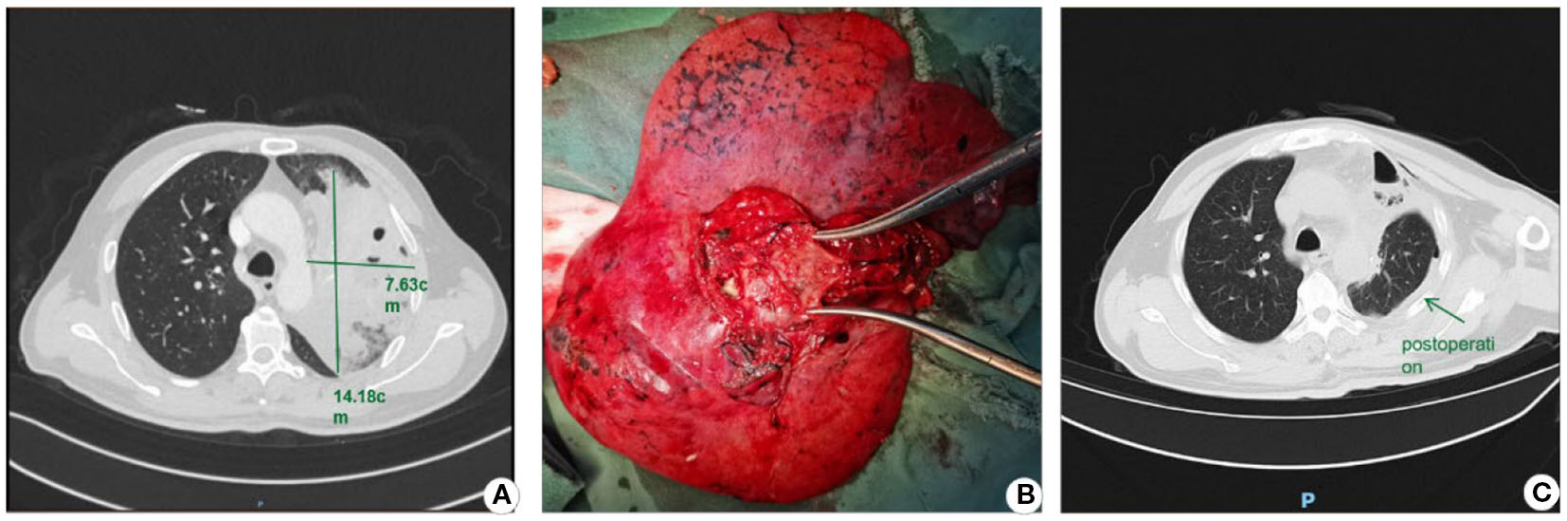
Lobectomy for lung cancer-associated lung abscess. **(A)** Preoperative CT image; **(B)** Resected lobe demonstrating the abscess cavity upon dissection; **(C)** Postoperative CT image.

A 51-year-old male was admitted to Sichuan Cancer Hospital on October 10, 2024, with a chief complaint of “non-small cell lung cancer (NSCLC) detected more than 20 days earlier, accompanied by productive cough and fever for 2 days.” PET-CT imaging revealed: 1. a soft tissue nodular mass in the left hilum with increased metabolism, consistent with lung cancer; 2. a mass in the anterior segment of the left upper lobe with ring-shaped metabolic activity, suggestive of a lung abscess; and 3. obstructive pneumonia in the left upper lobe. Laboratory tests on admission showed elevated inflammatory markers. Initial treatment with cefoperazone-sulbactam was ineffective, with persistent recurrent fever. Antibiotic therapy was escalated to imipenem-cilastatin plus vancomycin. Follow-up CT showed poor response to anti-infective therapy. Emergency radical resection of the left upper lobe lung cancer was performed on October 17, 2024, as shown in **Figure 3**. The procedure was successful, and previous antibiotic regimens were continued postoperatively. The patient recovered well, with normalized body temperature and inflammatory markers, and was discharged on October 24, 2024. Pathology confirmed left lung squamous cell carcinoma, pT3N1M0 Stage IIIA. This case highlights that bronchial obstruction relief is necessary when obstructive pneumonia is refractory to conventional management.

## Research advances and frontier technologies

Novel Diagnostic Technology: Metagenomic next-generation sequencing (mNGS) significantly enhances detection of polymicrobial coinfections (e.g., bacterial-fungal-viral) through unbiased pathogen nucleic acid analysis in bronchoalveolar lavage fluid (BALF), proving particularly valuable for immunocompromised patients (Chen S. et al., 2022). Droplet digital PCR (ddPCR) quantifies low-abundance resistance genes [e.g., blaKPC (Wu et al., 2024), ;mecA (Luo et al., 2017)] to guide precision antimicrobial therapy Concurrently, deep learning-based CT analysis systems (e.g., AI-Rad Companion) now automate detection of obstructive pneumonia features like mucoid impaction and abscess cavitation (Niehoff et al., 2023), while natural language processing (NLP) models integrate electronic health records with microbiological data to predict resistance risks and recommend personalized regimens (Mora et al., 2022).

Novel Therapeutic Technologies: Nanomedicine has demonstrated significant progress in anti-inflammatory applications, exhibiting unique advantages for modulating pulmonary inflammatory disorders (e.g., pneumonia, asthma, acute lung injury, and COVID-19-associated cytokine storms) (Constantin et al., 2021; Ahmad, 2022; Zoulikha et al., 2022; Hu et al., 2023). Detailed information can be found in **Table 3**. Clinically relevant nanocarriers include: lipid nanoparticles and albumin-based nanovehicles enabling targeted delivery of chemotherapeutic/anti-inflammatory agents (e.g., paclitaxel, dexamethasone) to precisely inhibit inflammatory pathways (Hegeman et al., 2011; Fu et al., 2022); monoclonal antibodies (e.g., tocilizumab) neutralizing key cytokines like IL-6 to control hyperinflammation in severe pneumonia (McElvaney et al., 2021); and investigational IL-4/IL-13 dual-acting vaccines with platelet-mimetic nanovehicles advancing long-term immunomodulation and precision targeting (Karo-Atar et al., 2018; Hu et al., 2023). Core advantages include: 1. Targeted delivery: Enhanced drug accumulation at inflammatory sites via surface modification or biomimetic design reduces systemic exposure [e.g., platelet vesicles targeting acute lung injury (Chen et al., 2023)]; 2. Stability and controlled release: Nanostructures protect payloads from degradation while enabling sustained or stimuli-responsive release [e.g., PLGA nanoparticles (Guan et al., 2024)]; Overcoming conventional limitations: Improved bioavailability of poorly soluble drugs [e.g., baicalein (Wang et al., 2015)] or direct alveolar delivery via inhalable formulations (e.g., meloxicam nanosuspensions (Party et al., 2022)]. Recent advances focus on combination therapies (anti-infection + immunomodulation) (Zhang et al., 2024), biomimetic carriers (e.g., cell membrane-coated nanoparticles) (Krishnan et al., 2024), and combatting drug-resistant infections (e.g., nanoformulated ceftazidime/avibactam) (Wang et al., 2022). Despite challenges in long-term safety evaluation and uniformity of pulmonary deposition, nanomedicine is emerging as a pivotal strategy for respiratory diseases through precise modulation of inflammatory microenvironments, with multidisciplinary convergence accelerating clinical translation.

**Table 3:** Clinically relevant and investigational nanomedicines: names and therapeutic areas.

Drug name	Therapeutic area	Stage	Brief introduction
Abraxane	Lung cancer	Launched	Albumin-bound paclitaxel nanoparticles enhancing therapeutic efficacy through improved drug delivery (Yardley, 2013).
Actemra	Severe COVID-19 (Cytokine Storm)	Launched	Tocilizumab (IL-6 inhibitor), blocking pro-inflammatory cytokines (McElvaney et al., 2021).
Fluenz Tetra	Influenza A/B	Launched	Live-attenuated vaccines (LAVs) stimulating mucosal immunity via intranasal administration (Thwaites et al., 2023).
IL-4/IL-13 dual-targeting vaccine	Allergic asthma	Experimental stage	Reduced eosinophil and IgE levels with long-term cytokine neutralization in mouse models (Conde et al., 2021).
Baicalein self-microemulsifying drug delivery system (SMEDDS)	Inflammatory lung diseases (such as COPD, acute lung injury)	Experimental stage	Significantly reduces levels of 14 cytokines including TNF-α and IL-6 following oral administration (Tang et al., 2024).
Meloxicam nanosuspension	Local pulmonary alveolar inflammation	Experimental stage	Inhalable formulation with 1.3 μm mass median aerodynamic diameter (MMAD), reducing IL-6 levels (Party et al., 2022).
Platelet-derived extracellular vesicles (PEVs)	Acute Lung Injury (ALI)	Experimental stage	Targeted delivery to inflammatory sites following intravenous administration, mitigating pulmonary tissue damage (Chen et al., 2023).

## Challenges and future perspectives

Lung cancer-associated obstructive pneumonia and pulmonary abscess management face two core challenges: First, balancing infection control with antineoplastic therapy toxicity. Broad-spectrum antibiotics may suppress infections but exacerbate chemotherapy/radiation-induced myelosuppression and mucosal injury, increasing secondary resistance risks. Second, compromised performance status (ECOG ≥2 or KPS ≤70) often precludes intensive therapies, forcing 30%-40% of patients into palliative approaches with significant survival implications.Future research priorities to optimize management include:Precision anti-infective/immunomodulatory integration: Rapid pathogen identification via metagenomic NGS (mNGS) to guide narrow-spectrum antibiotics, reducing resistance risks. Concurrently explore synergy between immune checkpoint inhibitors (ICIs) and antimicrobials—e.g., nano carrier-mediated local antibiotic delivery to minimize systemic toxicity, or ICI reintroduction post-infection control to delay tumor progression.Targeted antineoplastic-interventional synergy: Develop biodegradable stents with drug-eluting coatings (e.g., paclitaxel/anti-inflammatory nanoparticles) to relieve obstruction while suppressing local tumor growth and inflammation. For abscesses, investigate bronchoscopic localized antimicrobial sustained-release systems (e.g., lipid-encapsulated vancomycin) to enhance intralesional drug concentration.Personalized regimens for frail patients: Stratify by ECOG/KPS to implement low-intensity chemotherapy (e.g., metronomic dosing) with supportive care (e.g., G-CSF prophylaxis), or leverage nanotechnology (e.g., albumin-bound nanodrugs) to enhance targeting and reduce systemic exposure.Resistance/recurrence mitigation: Implement dynamic resistance gene surveillance; develop nanozyme or phage therapies against biofilm infections; optimize radiotherapy planning (e.g., SBRT) to minimize normal lung injury and secondary infection risks.Multidimensional data-driven decisions: Integrate radiomics, ctDNA, and inflammatory biomarkers (e.g., IL-6, PCT) to build predictive models dynamically assessing infection-tumor interplay, guiding therapeutic timing adjustments.Clinical translation of these strategies may overcome current limitations, achieving dual objectives of infection eradication and tumor control to ultimately improve survival quality and outcomes.
